# The association between short video addiction and emotion dysregulation among college students: a latent profile analysis and its influencing factors

**DOI:** 10.3389/fpsyg.2026.1789207

**Published:** 2026-03-13

**Authors:** Shuhe Wang, Zhongguo Liu

**Affiliations:** School of Journalism and Communication, Shandong Normal University, Jinan, China

**Keywords:** cognitive reappraisal, college students, emotion dysregulation, emotional loneliness, short video addiction

## Abstract

**Objective:**

This study aimed to use latent profile analysis (LPA) to identify heterogeneous configurational patterns of short video addiction and emotion dysregulation among college students, and to systematically examine the predictive effects of cognitive reappraisal, emotional loneliness, and sociodemographic factors on latent profile membership.

**Methods:**

A cross-sectional survey design was employed. From April to July 2025, full-time undergraduate students were recruited from multiple universities in Shandong Province using a combination of convenience sampling and snowball sampling. Participants completed online questionnaires including the Short Video Addiction Scale, the Emotion Dysregulation Inventory (EDI), the Cognitive Reappraisal Scale, and the Emotional Loneliness Scale.

**Results:**

A total of 1,168 valid questionnaires were obtained. LPA identified four optimal profiles: Profile 1 (“low short video addiction–low emotion dysregulation”), Profile 2 (“medium to lower short video addiction–medium to lower emotion dysregulation”), Profile 3 (“medium to upper short video addiction–medium to upper emotion dysregulation”), and Profile 4 (“high short video addiction–high emotion dysregulation”). Multivariable logistic regression analyses indicated that, with Profile 4 as the reference category, cognitive reappraisal significantly increased the likelihood of membership in lower-risk profiles, whereas emotional loneliness significantly decreased the likelihood of membership in lower-risk profiles. Among sociodemographic factors, being female and having an urban background significantly increased the likelihood of membership in Profile 1 (vs. Profile 4); being a non-only child and having no part-time work experience significantly predicted membership in Profile 3.

**Conclusion:**

Marked heterogeneity exists among college students in the measured dimensions of short-form video addiction and emotion dysregulation, and the two constructs exhibit highly concordant co-variation. The findings provide empirical support for developing risk-stratified and precision-oriented mental health intervention strategies.

## 1 Introduction

Short video platforms have become a central medium for contemporary youth to obtain information, engage in social interaction, and enjoy leisure activities (Lu et al., 2022; Zheng, 2023). According to statistical reports released by the China Internet Network Information Center, by 2024, the number of short video users in China has exceeded 1 billion, with college students aged 18 to 24 forming the most active user group (Yuan and Wang, 2024; Zhang and Zhu, 2025). These platforms, leveraging algorithm-driven recommendations, fragmented content delivery, and instant feedback systems, effectively cater to young users’ psychological needs for novelty, excitement, and immediate gratification (Nguyen et al., 2025). However, this highly immersive media experience has raised widespread concerns among scholars about the risk of behavioral addiction (Bojić et al., 2025). Short video addiction, as an emerging form of technological behavioral addiction, is characterized by compulsive dependence on short video use, increased uncontrollable usage, and negative emotional experiences during withdrawal (Li et al., 2025; Zhang et al., 2019). This addiction can significantly impair academic performance, interpersonal relationships, and mental health (Ye et al., 2022; Jiang et al., 2024). For college students, who are at a critical stage of psychological development, short video addiction may interfere not only with their academic engagement and career planning but also have profound negative effects on their emotional regulation abilities and overall mental well-being (Ding et al., 2024).

Emotion dysregulation refers to difficulties or impairments in recognizing, understanding, accepting, and regulating emotions, often manifesting as excessive or prolonged emotional responses and ineffective regulation strategies (Cole et al., 1994; Faraone et al., 2019). Previous studies have found strong links between emotion dysregulation and various psychopathologies, including depression, anxiety, borderline personality traits, and addictive behaviors (Chapman, 2019; Sideli et al., 2025). College students are in a transitional phase from adolescence to early adulthood, facing multiple developmental challenges such as academic pressure, social adaptation, identity formation, and career planning (Yi et al., 2024; Zhao et al., 2023). Their emotional regulation systems are under considerable strain (Demichelis et al., 2024). When lacking effective emotional regulation skills, individuals may turn to instant digital media like short videos to escape emotions or seek quick satisfaction, potentially leading to addictive use patterns (Singh et al., 2025). Conversely, excessive short video use may further impair emotional regulation by occupying cognitive resources, weakening real-life social interactions, and reinforcing tendencies toward immediate gratification, creating a vicious cycle (Zhang et al., 2023).

Traditional variable-centered research paradigms exploring the relationship between short video addiction and emotion dysregulation reveal overall correlational trends (Yao et al., 2025; Wang et al., 2023) but struggle to capture individual heterogeneity. For example, Wang, Wang (Wang et al., 2023) analyzed the longitudinal links between emotion dysregulation and short video addiction. However, college students likely exhibit multiple qualitatively different subgroups in their short video usage and emotion regulation patterns rather than a single uniform distribution. Latent profile analysis (LPA), a person-centered mixture modeling technique, can identify hidden heterogeneous subgroups within a sample based on multiple continuous indicators and provide probabilistic classification for each profile (Muthén and Muthén, 2000). In recent years, LPA has been widely applied in addiction and emotional problem research, successfully identifying different risk profiles among online gaming addicts, heterogeneous subtypes of depression symptoms, and combined patterns of emotion regulation strategies (Wang et al., 2024; Cerniglia et al., 2019). However, most existing studies perform latent class analysis on either short video addiction or emotion dysregulation alone, with few integrating both constructs into a unified latent profile analysis (Ding et al., 2024; Rufino et al., 2017; Blay et al., 2025). LPA enables simultaneous examination of co-varying patterns of short video addiction and emotion dysregulation, identifying subgroups with specific combined characteristics on both variables, thus providing a more comprehensive understanding of their complex relationship and heterogeneity.

Internal cognitive-emotional regulation strategies and social–emotional needs likely play key roles in predicting profile membership of short video addiction and emotion dysregulation. Cognitive reappraisal, an adaptive emotion regulation strategy, involves changing one’s interpretation of emotion-eliciting situations to modulate emotional responses (Cutuli, 2014; Wolgast et al., 2011). According to the emotion regulation process model, cognitive reappraisal is an antecedent-focused strategy occurring before full emotional activation, making it highly effective (Schutte et al., 2009). Empirical evidence shows that individuals who habitually use cognitive reappraisal experience fewer negative emotions, greater psychological resilience, and less problematic internet use (Senol-Durak and Durak, 2017). Therefore, the level of cognitive reappraisal ability may influence whether an individual falls into high-risk profiles for short video addiction and emotion dysregulation. Meanwhile, emotional loneliness, the subjective experience of lacking close emotional connections (Roberts and Krueger, 2021), plays an important role in the formation of digital media addiction. The social compensation hypothesis suggests that individuals with higher emotional loneliness are more inclined to seek emotional comfort and belonging through virtual social media (Cohen and Lancaster, 2014; Hall, 2025), and the quasi-social interaction provided by short video platforms meets this need, increasing addiction risk. Emotional loneliness is also inherently linked to emotional regulation difficulties, as individuals lacking intimate support systems tend to be more vulnerable when facing emotional challenges (Preece et al., 2021).

Sociodemographic characteristics may also serve as important predictors of profile membership in short video addiction and emotion dysregulation. Regarding gender, females generally show higher emotional expressiveness and social support seeking but are also more prone to emotion dysregulation symptoms (Shangguan et al., 2022), while males tend to exhibit higher risk for internet addiction behaviors (Sung et al., 2013). Across academic years, students face different developmental tasks and stressors, leading to varying short video use motives and emotional states. Family structure, as a source of early attachment experience and social support (Green et al., 2011), may influence emotional security and coping resources, thus associating with the studied variables. Family income reflects economic capital and access to material resources; economic stress itself is a risk factor for emotion dysregulation and may also affect media use habits. Differences in hometown locations may indicate variations in media exposure history and cultural values. Part-time job experience could influence short video use patterns by providing real-life social opportunities and structured time. Examining these sociodemographic factors together helps identify the external characteristics of high-risk subgroups and provides targeted directions for precise interventions.

Current research gaps include: (1) reliance on variable-centered paradigms that overlook individual-level heterogeneous combination patterns between short video addiction and emotion dysregulation; (2) scarcity of empirical latent profile analyses integrating both constructs; and (3) lack of systematic examination of key psychological variables such as cognitive reappraisal and emotional loneliness, alongside multiple sociodemographic predictors, in relation to profile membership. Based on these gaps, this study targets college students and applies latent profile analysis to first identify latent profile types of short video addiction and emotion dysregulation, then test how cognitive reappraisal and emotional loneliness predict profile membership, and finally systematically examine gender, academic year, only-child status, family income, hometown, part-time job status, romantic relationship status, and family structure differences across profiles. The study aims to deepen understanding of the short video addiction-emotion dysregulation relationship from a person-centered perspective, clarify psychological and social characteristics of high-risk subgroups, and provide evidence-based support for differentiated, precise mental health intervention strategies.

## 2 Research methods

### 2.1 Study design

This study used a cross-sectional survey design to explore the latent profile types of short video addiction and emotion dysregulation among college students, as well as their influencing factors. A cross-sectional design allows for systematic collection of large-sample data at a specific point in time, making it suitable for describing associations between variables and identifying heterogeneous subtypes within a population (Wang and Cheng, 2020).

### 2.2 Participants

#### 2.2.1 Recruitment procedure

Participants were recruited between April and July 2025 using a combination of convenience and snowball sampling. First, the researchers obtained approval from class counselors from three universities in Shandong Province (anonymized as U1–U3 to protect institutional privacy where requested) by explaining the study’s purpose and benefits. Two recruitment channels were used: (1) university-based distribution (e.g., class groups or student affairs announcements) and (2) open online distribution (social media posts and respondent-driven link forwarding/snowballing). Specifically, invitations were sent to enrolled undergraduates. Recruitment was further expanded by posting announcements on social media platforms. Finally, participants were encouraged to share the survey link with their peers via snowball sampling. To ensure sample representativeness and diversity, recruitment targeted students from different types of universities and academic years.

The study strictly adhered to the ethical principles outlined in the Declaration of Helsinki. The research protocol was approved by the Academic Ethics Committee of Shandong Normal University. All potential participants were required to read an electronic informed consent form before completing the questionnaire, clearly explaining the study’s purpose, procedures, confidentiality, voluntary participation, and the right to withdraw at any time. Only after agreeing were participants allowed to proceed to the formal survey. The survey was administered via a professional online platform, Credamo^1^. To protect privacy and data security, responses were collected anonymously without recording any personally identifiable information.

#### 2.2.2 Inclusion and exclusion criteria

Inclusion criteria were: (1) full-time undergraduate students aged 18–25; (2) ownership of a smartphone and experience using short video platforms; (3) ability to independently read and understand the Chinese questionnaire; (4) voluntary participation with signed electronic informed consent.

Exclusion criteria were: (1) currently receiving psychiatric medication or psychological therapy; (2) survey completion time either too short (less than one-third of the median time) or too long (more than three times the median), suggesting careless or distracted responses; (3) failure on embedded attention-check questions; (4) obvious response patterns or incomplete questionnaire data. Participants currently receiving psychiatric medication or psychological therapy were excluded to reduce clinical-treatment–related confounding (e.g., symptom stabilization or treatment-induced changes in emotion regulation) and to better characterize latent profiles in a non-clinical undergraduate population.

#### 2.2.3 Minimum sample size

The required sample size for latent profile analysis depends on the number of indicator variables, expected number of profiles, and the degree of separation between classes. According to Monte Carlo simulation studies, when using 2 to 4 indicator variables and expecting 3–5 profiles, the minimum sample size should be between 300 and 500 to ensure stable parameter estimation and accurate classification (Tein et al., 2013). Additionally, considering multivariate logistic regression, G*Power software was used to estimate a minimum sample size of 172, with an effect size of 0.15, alpha = 0.05, and power (1-*β*) = 0.95.

#### 2.2.4 Final sample

**Figure 1:**
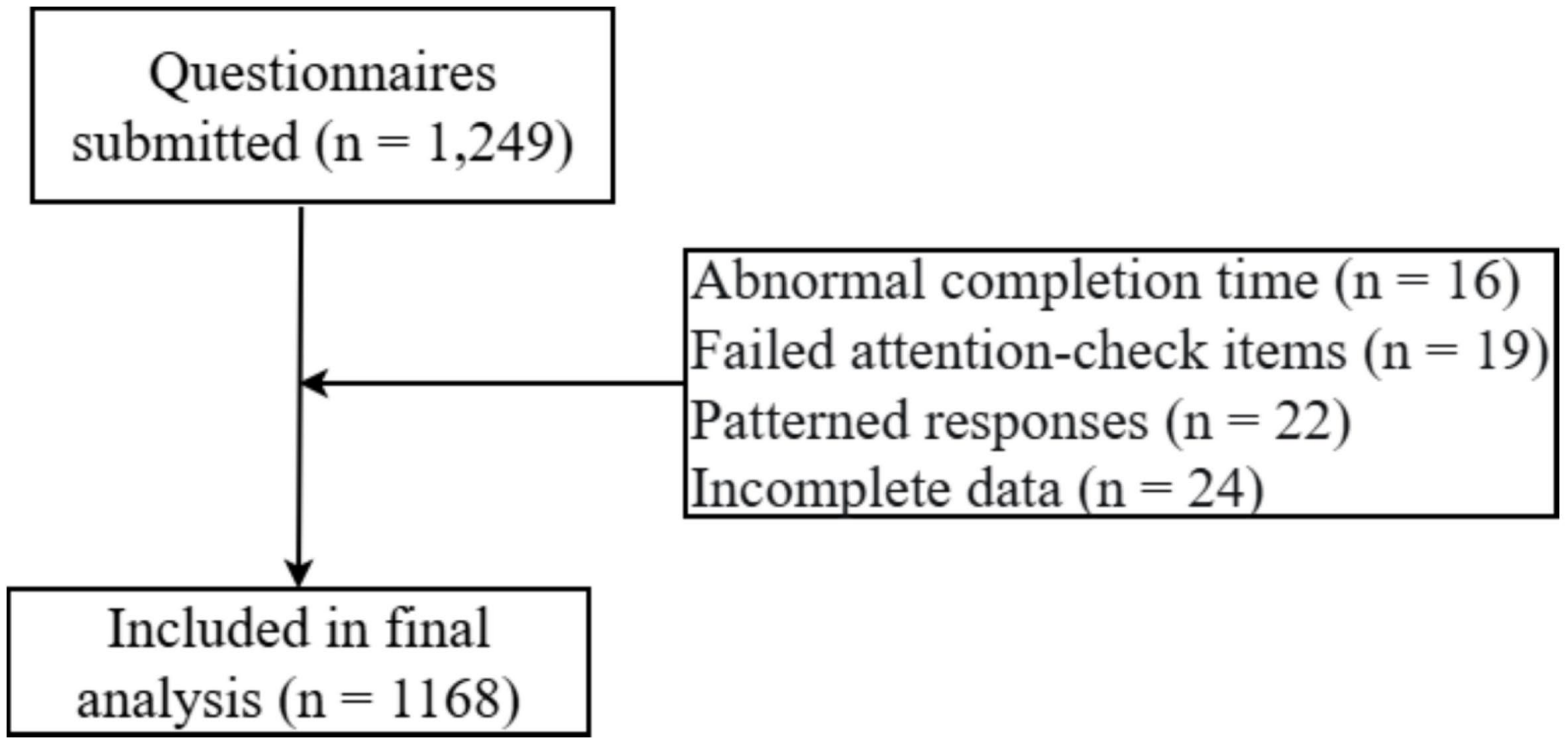
STROBE flow diagram of participant recruitment and inclusion.

A total of 1,249 questionnaires were submitted. After data cleaning, 81 questionnaires were excluded due to abnormal completion time (*n* = 16), failing attention-checks items (*n* = 19), patterned responses (*n* = 22), or incomplete data (*n* = 24). The final valid sample included 1,168 questionnaires, with an effective response rate of 93.51%, as shown in Figure 1.

### 2.3 Measurement instruments

#### 2.3.1 Short video addiction scale

The study used the Chinese version of the Short Video Addiction Scale developed by Ye et al. (2025) to assess addiction severity among college students. This scale covers dimensions such as academic procrastination, interpersonal conflict, social communication difficulties, attention problems, and use impairment, with 15 items total. It uses a 5-point Likert scale from “Never = 1” to “Always = 5,” summing item scores to produce a total score, where higher scores indicate more severe addiction. For example, “Watching short videos makes me increasingly impatient with other activities such as conversations or reading.” In this study’s sample, the scale showed excellent internal consistency with a Cronbach’s α of 0.953, indicating strong reliability among college students.

#### 2.3.2 Emotion dysregulation inventory

Emotion dysregulation was measured using the Emotion Dysregulation Inventory (EDI), which assesses difficulties in emotional reactivity and regulation (Mazefsky et al., 2018). Originally developed for autism spectrum disorder populations, the EDI has since been validated for general populations. The EDI includes two core dimensions: Reactivity (24 items) and Dysregulation (6 items), using a 5-point Likert scale from “Not at all true = 1” to “Completely true = 5.” Total and subscale scores are summed, with higher scores indicating greater emotion dysregulation. Kuo et al. (2025) translated and validated the Chinese version, confirming its cultural suitability and reliability. In this study, the Cronbach’s *α* was 0.921, demonstrating good reliability.

#### 2.3.3 Cognitive reappraisal scale

The Cognitive Reappraisal subscale from the Emotion Regulation Questionnaire (ERQ) (Gross and John, 2003) was used to assess habitual use of cognitive reappraisal strategies among students. This subscale contains 6 items measuring the tendency to regulate emotions by changing the way one thinks about emotional situations, e.g., “When I want to feel less negative emotion, I change the way I think about the situation.” Items are rated on a 5-point Likert scale from “Strongly disagree = 1” to “Strongly agree = 5,” with scores averaged to produce a subscale score; higher scores indicate greater use of cognitive reappraisal. The Chinese version, translated by Li et al. (2018), has been validated for cultural appropriateness and reliability in Chinese college populations. The Cronbach’s *α* in this study was 0.866.

#### 2.3.4 Emotional loneliness scale

Emotional loneliness was measured using the Emotional Loneliness subscale of the Short-form Social and Emotional Loneliness Scale for Adults (SELSA-S) (DiTommaso et al., 2004). Emotional loneliness arises from lack of intimate attachment and can be divided into family emotional loneliness and romantic emotional loneliness. This subscale includes 10 items covering both dimensions, such as “I feel a lack of closeness with my family” and “I long for a close romantic relationship.” Responses use a 5-point Likert scale from “Strongly disagree” (Lu et al., 2022) to “Strongly agree” (Nguyen et al., 2025), with some items reverse scored. Higher scores indicate greater emotional loneliness. The scale has been widely used and validated for Chinese college students (Chen et al., 2024). The Cronbach’s *α* in this study was 0.893.

### 2.4 Statistical analysis

Data analysis was conducted using SPSS 27.0 and Mplus 8.3. Internal consistency reliability of scales was evaluated using Cronbach’s α (acceptable if *α* > 0.70). Harman’s single-factor test was used to check for common method bias, with a threshold of 40%. Descriptive statistics and correlation analyses examined relationships, means, and distributions of short video addiction, emotion dysregulation, cognitive reappraisal, and emotional loneliness.

Latent Profile Analysis (LPA): Scores on dimensions of short video addiction and emotion dysregulation were used as indicator variables to fit models ranging from 1 to 5 profiles. Model fit was assessed using multiple indices: Akaike Information Criterion (AIC), Bayesian Information Criterion (BIC), and adjusted BIC (aBIC), with lower values indicating better fit; entropy (values > 0.8) indicating classification accuracy; Lo–Mendell–Rubin likelihood ratio test (LMR-LRT) and bootstrap likelihood ratio test (BLRT), where *p* < 0.05 suggests the k-profile model fits significantly better than the k-1 profile model. Chi-square tests and one-way ANOVA were used to examine differences in sociodemographic variables across profiles and to compare cognitive reappraisal and emotional loneliness scores among profiles. Finally, multivariate logistic regression analyzed predictors of profile membership, reporting odds ratios (OR) and 95% confidence intervals.

LPA was conducted in Mplus 8.3 using TYPE = MIXTURE with the MLR estimator. We used STARTS = 200 50 with PROCESSORS = 4, requested TECH11 (LMR) and TECH14 (BLRT) for model comparison, and confirmed that the best log-likelihood was replicated, as shown in Supplementary methods.

## 3 Results

### 3.1 Demographic information

The study included 615 male participants (52.70%) and 553 female participants (47.30%). Most participants were sophomores (*N* = 393, 33.60%) and juniors (*N* = 391, 33.50%). Non-only children accounted for 591 participants (50.60%). The majority of families reported a monthly income between 6,000 and 9,000 RMB (*N* = 550, 47.10%). Detailed demographic information is presented in Table 1.

**Table 1:** Demographic information of the participants.

Variables	Items	Number (*N*)	Proportion (%)
Gender	Male	615	52.70%
	Female	553	47.30%
Grade	Freshman	190	16.30%
	Sophomore	393	33.60%
	Junior	391	33.50%
	Senior	194	16.60%
Only children	No	591	50.60%
	Yes	577	49.40%
Monthly income	6,000 RMB and below	321	27.50%
	6,001–9,000 RMB	550	47.10%
	9,001 RMB and above	297	25.40%
Region of origin	Urban	585	50.10%
	Rural	583	49.90%
Part-time job	No	575	49.20%
	Yes	593	50.80%
Relationship status	Single	595	50.90%
	In a relationship	573	49.10%

### 3.2 Common method bias

Since all variables were measured via self-report questionnaires, there was potential for common method bias due to consistency motifs or social desirability. To address this, participants were informed that there were no right or wrong answers to reduce social desirability bias. Validated scales with reverse-scored items were used to avoid ambiguous wording. The anonymity and confidentiality of responses were emphasized.

Harman’s single-factor test was conducted by including all items in an exploratory factor analysis using principal component extraction for factors with eigenvalues greater than 1. The first common factor explained 24.474% of the variance, below the 40% threshold, indicating no serious common method bias in the data.

### 3.3 Descriptive statistics and correlations

Descriptive statistics and correlations of the core study variables are shown in Tables 2, 3. Scores for short video addiction (*M* = 2.647, SD = 0.699), emotion dysregulation (*M* = 2.959, SD = 0.456), cognitive reappraisal (*M* = 2.993, SD = 0.574), and emotional loneliness (*M* = 2.995, SD = 0.560) were all below the midpoint value of 3. Skewness ranged from −0.268 to 0.085 and kurtosis from 0.003 to 0.315, indicating acceptable normality (|Skewness| ≤ 3, |Kurtosis| ≤ 10).

**Table 2:** Descriptive statistics of core variables.

Variables	*M*	SD	Skewness	Kurtosis
Short video addiction	2.647	0.699	−0.268	0.111
Emotion dysregulation	2.959	0.456	0.085	0.315
Cognitive reappraisal	2.993	0.574	−0.083	0.274
Emotional loneliness	2.995	0.560	−0.067	0.003

M, mean; SD, standard deviation.

**Table 3:** Correlation analysis of core variables.

Variables	1	2	3	4
1. Short video addiction	1			
2. Emotion dysregulation	0.480***	1		
3. Cognitive reappraisal	−0.126***	−0.303***	1	
4. Emotional loneliness	0.116***	0.373***	−0.271***	1

***p < 0.001.

Correlation analysis found a significant moderate positive correlation between short video addiction and emotion dysregulation (*r* = 0.480, *p* < 0.001), meaning higher addiction was associated with greater emotion dysregulation. Short video addiction showed a weak positive correlation with emotional loneliness (*r* = 0.116, *p* < 0.001) and a weak negative correlation with cognitive reappraisal (*r* = −0.126, *p* < 0.001). Emotion dysregulation was weakly positively correlated with emotional loneliness (*r* = 0.373, *p* < 0.001) and weakly negatively correlated with cognitive reappraisal (*r* = −0.303, *p* < 0.001). Cognitive reappraisal was weakly negatively correlated with emotional loneliness (*r* = −0.271, *p* < 0.001). Correlations ranged between −0.303 and 0.480, well below the 0.85 threshold for multicollinearity concerns.

### 3.4 Latent profile analysis of short video addiction and emotion dysregulation

#### 3.4.1 Determining the optimal number of profiles

Using dimensions of short video addiction and emotion dysregulation as indicators, models with 1–5 latent profiles were fitted. Fit indices for each model are shown in Table 4. As profile number increased, AIC, BIC, and adjusted BIC values decreased but with diminishing improvements. Entropy values for 2–5 profile models all exceeded the acceptable 0.80 threshold, with the 4-profile model having the highest entropy (0.920), indicating optimal classification accuracy. Likelihood ratio tests (LMR-LRT and BLRT) for models with 2–5 profiles all had *p*-values below 0.05, showing each model significantly improved fit over the previous one. However, the 5-profile model’s entropy was slightly lower than the 4-profile model’s, and its smallest profile proportion was only 4.2%, close to the 5% minimum threshold, raising concerns about stability. The 4-profile model revealed clear, theoretically meaningful distinctions across short video addiction and emotion dysregulation dimensions. Therefore, the 4-profile model was selected as optimal.

**Table 4:** Fit indices for each profile.

Profile	AIC	BIC	aBIC	Entropy	LMR (p)	BLRT (p)	Smallest proportion per class				
							1	2	3	4	5
1	17146.459	17217.342	17172.873	-	-	-	-				
2	14246.977	14358.364	14288.484	0.882	<0.001	<0.001	40.7%	59.3%			
3	13019.720	13171.611	13076.321	0.914	<0.001	<0.001	42.3%	9.7%	47.9%		
**4**	**12058** **.442**	**12250.837**	**12130.136**	**0.920**	**<0.001**	**<0.001**	**9.2%**	**44.7%**	**36.0%**	**10.1%**	
5	11865.693	12098.593	11952.482	0.890	0.003	<0.001	9.0%	33.0%	38.8%	14.9%	4.2%

Note: Bold text indicates the optimal fitting profile.

#### 3.4.2 Profile characteristics

**Figure 2:**
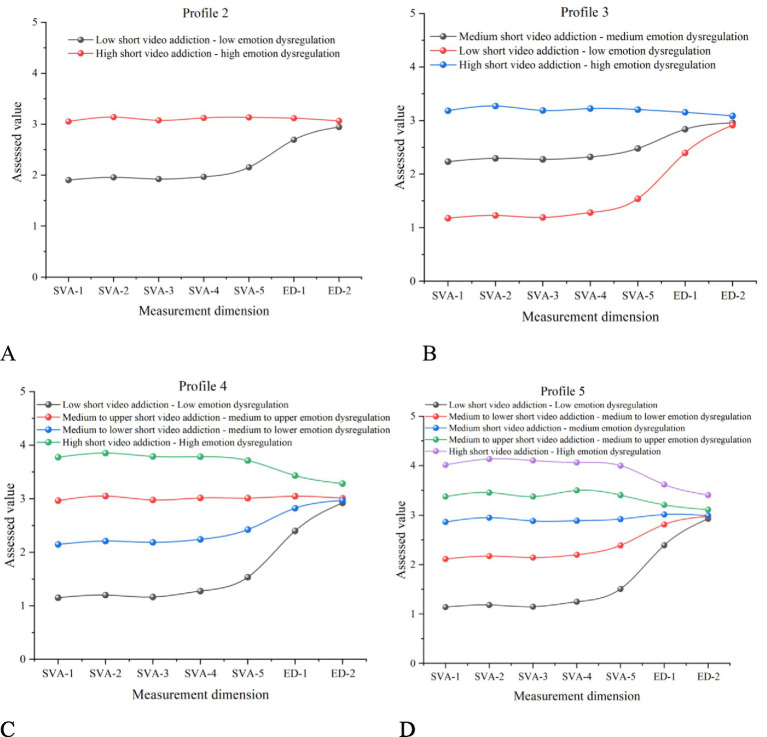
Profile characteristics across the four latent profiles. **(A)** 2 Latent subgroup profile plots; **(B)** 3 Latent subgroup profile plots; **(C)** 4 Latent subgroup profile plots; **(D)** 5 Latent subgroup profile plots.

Profile characteristics were plotted using Origin 2021 software (see Figure 2, with the optimal solution shown as Figure 2C).

Based on relative levels across the two dimensions, the four profiles were named as follows: Profile 1, representing a low-risk subgroup (“Low short video addiction – Low emotion dysregulation”), comprised 9.2% of the sample (*N* = 108). These students demonstrated reasonable control over short video use and good emotional regulation, reflecting the best overall psychological adjustment.

Profile 2, the moderately low-risk group (“Medium to lower short video addiction – medium to lower emotion dysregulation”), comprised 36.0% of participants (*N* = 420), displaying moderately low scores on both dimensions and relatively good adjustment, though continued attention is warranted to prevent risk escalation.

Profile 3, labeled the moderately high-risk group (“Medium to upper short video addiction – medium to upper emotion dysregulation”), accounted for 44.7% (*N* = 522), representing the largest subgroup. These students exhibited moderate levels of distress on both dimensions, indicating a need for psychological health monitoring and early intervention.

Profile 4, the high-risk group (“High short video addiction – high emotion dysregulation”), included 10.1% of the sample (*N* = 118) and exhibited the highest levels on both dimensions, representing a comorbid high-risk population warranting prioritized mental health resources.

### 3.5 Differences among latent profiles

Differences in demographic variables across the four profiles were examined using chi-square tests, while one-way ANOVA tested differences in cognitive reappraisal and emotional loneliness (Table 5). Significant differences emerged for gender (*χ*^2^ = 9.496, *p* = 0.023), with females more prevalent in the low-risk profile (Profile 1) and males predominating the other profiles. Only-child status differed significantly (*χ*^2^ = 15.855, *p* = 0.001), with non-only children more common in the moderately high-risk group (Profile 3). Family monthly income varied significantly (*χ*^2^ = 16.105, *p* = 0.013), with middle-income families (6,000–9,000 RMB) most represented. Place of origin was significant (*χ*^2^ = 14.251, *p* = 0.003); rural students were more frequent in the moderately high-risk and high-risk groups. Part-time job experience differed significantly (*χ*^2^ = 12.934, *p* = 0.005), with those holding part-time jobs more common in the high-risk profile. Relationship status also varied (*χ*^2^ = 13.101, *p* = 0.004), with single students predominating the moderately low-risk group. No significant difference was found for grade level (*χ*^2^ = 7.282, *p* = 0.608), indicating limited influence on profile membership.

**Table 5:** Single-factor analysis of demographic differences across profiles of short video addiction and emotion dysregulation.

Variables	Items	Profile 1	Profile 2	Profile 3	Profile 4	*χ*^2^/*F*	*p*
Gender	Male	44	217	283	71	9.496	0.023
	Female	64	203	239	47		
Grade	Freshman	23	63	88	16	7.282	0.608
	Sophomore	40	140	174	39		
	Junior	29	142	173	47		
	Senior	16	75	87	16		
Only children	No	43	197	296	55	15.855	0.001
	Yes	65	223	226	63		
Monthly income	6,000 RMB and below	32	95	168	26	16.105	0.013
	6,000–9,000 RMB	48	223	220	59		
	9,001 RMB and above	28	102	134	33		
Region of Origin	Urban	69	222	237	57	14.251	0.003
	Rural	39	198	285	61		
Part-time job	No	43	203	282	47	12.934	0.005
	Yes	65	217	240	71		
Relationship status	Single	44	241	252	58	13.101	0.004
	In a relationship	64	179	270	60		
Cognitive reappraisal		3.086 ± 0.555	3.058 ± 0.582	2.969 ± 0.557	2.782 ± 0.587	8.456	<0.001
Emotional loneliness		2.923 ± 0.566	2.945 ± 0.576	2.992 ± 0.537	3.247 ± 0.530	9.92	<0.001

Profile 1 = Low short video addiction – low emotion dysregulation; Profile 2 = Medium to lower short video addiction – medium to lower emotion dysregulation; Profile 3 = Medium to upper short video addiction – medium to upper emotion dysregulation; Profile 4 = High short video addiction – high emotion dysregulation.

Regarding psychological variables, cognitive reappraisal scores differed significantly across profiles (*F* = 8.456, *p* < 0.001). The low-risk profile (Profile 1) had the highest cognitive reappraisal mean (*M* = 3.086, SD = 0.555), whereas the high-risk profile (Profile 4) had the lowest (*M* = 2.782, SD = 0.587). This suggests an inverse association between cognitive reappraisal ability and risk level, with lower reappraisal linked to higher risk. Emotional loneliness also varied significantly (*F* = 9.920, *p* < 0.001), with Profile 4 showing the highest loneliness (*M* = 3.247, SD = 0.530) and Profile 1 the lowest (*M* = 2.923, SD = 0.566), indicating a positive relationship between loneliness and risk level. Profile-specific means and standard deviations for the indicator variables and the distal variables (cognitive reappraisal and emotional loneliness) are provided in Supplementary Table S1.

### 3.6 Multivariate logistic regression analysis

Profile 4 was used as the reference category; therefore, odds ratios (OR) greater than 1 indicate higher odds of being classified into Profiles 1–3 rather than Profile 4.

A multivariate logistic regression was conducted to explore independent predictors of profile membership, using Profile 4 as the reference group and including cognitive reappraisal, emotional loneliness, gender, only-child status, family income, place of origin, part-time job experience, and relationship status as predictors (Table 6). Compared to Profile 4, Profile 1 membership was significantly positively predicted by cognitive reappraisal (*B* = 0.680, OR = 1.974, 95% CI = [1.216, 3.207], *p* = 0.006), indicating each one-point increase in reappraisal nearly doubled the odds of belonging to Profile 1 rather than Profile 4. Emotional loneliness was a significant negative predictor (*B* = −0.887, OR = 0.412, 95% CI = [0.249, 0.682], *p* = 0.001), with higher loneliness reducing the odds of Profile 1 membership by 58.8%. Gender was also significant (*B* = −0.844, OR = 0.430, 95% CI = [0.249, 0.742], *p* = 0.002), with males 57% less likely than females to be in Profile 1. Place of origin was significant (*B* = 0.663, OR = 1.940, 95% CI = [1.123, 3.350], *p* = 0.017), with urban students nearly twice as likely as rural students to be in Profile 1.

**Table 6:** Multivariate logistic analysis of differences across the four latent profiles of short video addiction and emotion dysregulation.

Profile	Variables	Items	*B*	SE	Wald *χ*^2^	P	OR	LLCI	ULCI
Profile 1	Cognitive reappraisal		0.680	0.247	7.558	0.006	1.974	1.216	3.207
	Emotional loneliness		−0.887	0.257	11.872	0.001	0.412	0.249	0.682
	Gender	Male	−0.844	0.278	9.204	0.002	0.43	0.249	0.742
		Female (refer)							
	Only children	No	−0.225	0.276	0.66	0.416	0.799	0.465	1.373
		Yes (refer)							
	Monthly income	6,000 RMB and below	0.364	0.378	0.928	0.335	1.439	0.686	3.017
		6,000–9,000 RMB	−0.007	0.331	0.00	0.983	0.993	0.519	1.898
		9,001 RMB and above (refer)							
	Region of Origin	Urban	0.663	0.279	5.651	0.017	1.94	1.123	3.35
		Rural (refer)							
	Part-time job	No.	−0.135	0.28	0.231	0.63	0.874	0.505	1.513
		Yes (refer)							
	Relationship status	Single	−0.315	0.275	1.309	0.253	0.73	0.426	1.252
		In a relationship (refer)							
Profile 2	Cognitive reappraisal		0.646	0.195	10.939	0.001	1.907	1.301	2.796
	Emotional loneliness		−0.829	0.204	16.449	0.001	0.436	0.292	0.652
	Gender	Male	−0.352	0.218	2.606	0.106	0.703	0.459	1.078
		Female (refer)							
	Only children	No	0.076	0.215	0.124	0.724	1.079	0.708	1.643
		Yes (refer)							
	Monthly income	6,000 RMB and below	0.103	0.305	0.113	0.737	1.108	0.609	2.015
		6,000–9,000 RMB	0.206	0.255	0.653	0.419	1.229	0.746	2.024
		9,001 RMB and above (refer)							
	Region of Origin	Urban	0.197	0.214	0.843	0.359	1.217	0.8	1.852
		Rural (refer)							
	Part-time job	No.	0.299	0.218	1.878	0.171	1.348	0.879	2.066
		Yes (refer)							
	Relationship status	Single	0.36	0.214	2.832	0.092	1.434	0.942	2.182
		In a relationship (refer)							
Profile 3	Cognitive reappraisal		0.393	0.189	4.324	0.038	1.481	1.023	2.145
	Emotional loneliness		−0.74	0.2	13.66	0.001	0.477	0.322	0.706
	Gender	Male	−0.248	0.213	1.353	0.245	0.78	0.514	1.185
		Female (refer)							
	Only children	No	0.467	0.21	4.959	0.026	1.595	1.058	2.406
		Yes (refer)							
	Monthly income	6,000 RMB and below	0.379	0.292	1.682	0.195	1.461	0.824	2.59
		6,000–9,000 RMB	−0.098	0.249	0.155	0.694	0.907	0.557	1.477
		9,001 RMB and above (refer)							
	Region of Origin	Urban	−0.121	0.209	0.333	0.564	0.886	0.588	1.335
		Rural (refer)							
	Part-time job	No.	0.552	0.213	6.751	0.009	1.737	1.145	2.635
		Yes (refer)							
	Relationship status	Single	−0.034	0.209	0.027	0.87	0.966	0.642	1.455
		In a relationship (refer)							

B = unstandardized regression coefficient; SE = standard error; Wald χ^2^ = Wald chi-square statistic; P = *p*-value; OR = odds ratio; LLCI and ULCI = lower and upper limits of the 95% confidence interval for the odds ratio. Profile 1 = Low short video addiction – low emotion dysregulation; Profile 2 = Medium to lower short video addiction – medium to lower emotion dysregulation; Profile 3 = Medium to upper short video addiction – medium to upper emotion dysregulation; Profile 4 = High short video addiction – high emotion dysregulation.

Comparing Profile 2 to Profile 4 revealed the strongest cognitive reappraisal effect (B = 0.646, OR = 1.907, 95% CI = [1.301, 2.796], *p* = 0.001), nearly doubling odds per point increase, and a significant negative effect of emotional loneliness (*B* = −0.829, OR = 0.436, 95% CI = [0.292, 0.652], *p* = 0.001). None of the demographic predictors reached statistical significance in this comparison.

When comparing Profile 3 to Profile 4, cognitive reappraisal again positively predicted membership (*B* = 0.393, OR = 1.481, 95% CI = [1.023, 2.145], *p* = 0.038), and emotional loneliness negatively predicted membership (*B* = −0.740, OR = 0.477, 95% CI = [0.322, 0.706], *p* = 0.001). Only-child status was significant (*B* = 0.467, OR = 1.595, 95% CI = [1.058, 2.406], *p* = 0.026), with non-only children 59.5% more likely to be in Profile 3. Part-time job experience was also significant (*B* = 0.552, OR = 1.737, 95% CI = [1.145, 2.635], *p* = 0.009), with students without part-time jobs 73.7% more likely to be in Profile 3.

In summary, cognitive reappraisal functions as a protective factor that significantly increases the likelihood of belonging to lower-risk profiles, particularly the low-risk profile. Emotional loneliness acts as a risk factor, significantly decreasing the likelihood of low-risk profile membership in a dose–response manner. Among demographic variables, male gender and rural origin independently increase risk for the high-risk profile, while urban origin, absence of part-time job experience, and non-only-child status show significant associations with specific profile memberships, providing empirical guidance for identifying priority populations for intervention.

## 4 Discussion

### 4.1 Latent profile characteristics of short video addiction and emotion dysregulation

This study identified a four-class latent profile model as the optimal solution, providing empirical evidence that college students do not exhibit a homogeneous distribution across the measurement dimensions of short video addiction and emotion dysregulation. Instead, significant individual differences and subgroup heterogeneity exist. This finding aligns with previous research on technological behavioral addictions, such as internet addiction and smartphone dependence, which also reveal heterogeneous subgroup patterns (Baggio et al., 2018; Lee et al., 2018). It further supports the unique value of a person-centered research approach in understanding complex psychological phenomena.

Specifically, the four profiles exhibit a clear gradient pattern across the core dimensions of short video addiction and emotion dysregulation. Profile 1, the “Low short video addiction – low emotion dysregulation” group, comprises 9.2% of the sample and represents the subgroup with the most optimal functional adaptation. Profile 4, the “High short video addiction – high emotion dysregulation” group, accounts for 10.1% and represents a high-risk subgroup facing dual challenges. The relatively small proportions of these two extreme profiles are consistent with the distribution patterns of psychopathological phenomena in general populations. Notably, Profile 3, the “Medium to upper short video addiction – Medium to upper emotion dysregulation” group, is the largest subgroup at 44.7%. This indicates that nearly half of the college students already show signs of functional impairment in short video use behaviors and emotional regulation, highlighting a substantial developmental risk that cannot be overlooked.

The co-variation pattern between short video addiction and emotion dysregulation across the four profiles is highly consistent: high short video addiction levels co-occur with high emotion dysregulation, while low short video addiction levels co-occur with low emotion dysregulation. This finding offers individual-centered empirical support for the intrinsic link between these constructs. According to the self-medication hypothesis of emotion regulation, individuals with emotion dysregulation may use short video consumption as a compensatory strategy to cope with negative emotions, temporarily alleviating emotional distress through immediate sensory stimulation and emotional escape (Squires et al., 2021; Zsido et al., 2021). The algorithm-driven recommendations and fragmented content format provided by short video platforms perfectly cater to emotionally vulnerable individuals’ psychological needs for instant gratification and cognitive relief, thereby increasing the risk of addictive use (Liao, 2024). Excessive short video consumption may further impair emotional regulation by depleting limited cognitive control resources, weakening delayed gratification capacity, and reducing opportunities for real-life social interaction, creating a vicious cycle (Xia et al., 2023). This bidirectional, reinforcing dynamic results in an intertwined and mutually exacerbating relationship between these two problem dimensions.

The four-profile structure in this study differs in some respects from previous latent class analyses focusing on single constructs. Earlier studies typically identified profiles characterized by either “high short video addiction” or “high emotion dysregulation” alone, without comparing the two together. Our unique findings likely reflect the high covariation between short video addiction and emotion dysregulation within the university student population. These two dimensions may share common underlying susceptibility factors such as impulsivity traits, executive function deficits, or early adverse attachment experiences. However, methodological factors such as sample characteristics or measurement instruments may also influence profile structure, warranting cross-validation in diverse samples and cultural contexts in future research.

### 4.2 Influencing factors of latent profile membership

#### 4.2.1 Protective role of cognitive reappraisal

Cognitive reappraisal significantly increases the likelihood of belonging to lower-risk profiles, showing the strongest protective effect in comparisons between Profile 1 and Profile 4. This finding aligns with theoretical expectations from the emotion regulation process model (Webb et al., 2012) and supports cognitive reappraisal as an adaptive strategy critical in preventing technological behavioral addictions and emotion dysregulation (Zsido et al., 2021; Extremera et al., 2019). Specifically, individuals who habitually use cognitive reappraisal can reduce emotional intensity by altering their cognitive interpretation of situations before emotional responses fully activate, thereby lessening the need to rely on external media for emotional escape (Milyavsky et al., 2019). These individuals are more likely to adopt problem-focused coping strategies rather than retreating into digital media immersion when facing stress. Furthermore, enhanced cognitive reappraisal ability is closely related to metacognitive monitoring, psychological flexibility, and cognitive complexity—higher-order cognitive functions that themselves constitute important protective factors against addictive behaviors (Marciniak et al., 2024). Notably, cognitive reappraisal also showed a significant protective effect when comparing Profile 2 and Profile 4, indicating that even for subgroups with relatively moderate risk, stronger reappraisal skills reduce the likelihood of progressing to high-risk profiles. This provides empirical support for implementing cognitive reappraisal training interventions across different risk levels, suggesting that enhancing reappraisal ability may have universal protective value for the entire university student population.

#### 4.2.2 Risk role of emotional loneliness

Emotional loneliness significantly decreases the likelihood of belonging to low-risk profiles, suggesting that the lack of intimate emotional connections may drive individuals to seek compensatory emotional comfort through virtual digital media, thereby increasing the risk of addictive use (Sbarra et al., 2019). Emotional loneliness itself is inherently linked to difficulties in emotion regulation. Individuals lacking stable, intimate attachment relationships often fail to develop effective emotion regulation skills during early development, and their internal working models may contain negative beliefs about self-worth and interpersonal trust, affecting their ability and manner of coping with negative emotions. Moreover, the quasi-social interactive experiences offered by short video platforms can partially satisfy lonely individuals’ emotional belonging needs. The virtual companionship felt while watching short videos, the one-way emotional bonds formed with content creators, and the community atmosphere in comment sections all provide temporary emotional relief for lonely individuals. However, such substitute satisfaction is often insufficient to fill the void left by real intimate relationships and may even exacerbate loneliness by reducing opportunities for real social interaction and skill practice.

#### 4.2.3 Differential impact of sociodemographic factors

Gender significantly predicts profile membership, with males being significantly less likely to belong to the low-risk profile compared to females. This partially aligns with previous findings on gender differences in internet addiction risk and suggests that male college students may be more vulnerable to the comorbid risk of short video addiction and emotion dysregulation (Wang et al., 2023). This vulnerability is mainly due to males’ typically higher sensation-seeking tendencies and impulsivity traits, both closely linked to addiction risk (van Dongen et al., 2022). Meanwhile, traditional gender role socialization, which discourages emotional expression among males, may limit their opportunities to develop adaptive emotion regulation strategies, increasing their risk of emotion dysregulation.

Place of origin also significantly predicts profile membership, with students from urban areas far more likely to belong to low-risk profiles than their rural counterparts. This urban–rural disparity likely reflects systemic inequalities in digital literacy, media usage habits, and access to mental health resources. Rural students may have had limited media exposure and guidance before entering university, making them more prone to uncontrolled use of abundant digital media resources upon entering higher education.

Only-child status and part-time job experience mainly predicted differences between Profile 3 and Profile 4. Non-only children were more likely to be classified into Profile 3 than only children, possibly reflecting complex factors such as sibling competition, resource allocation, and diluted parental supervision in multi-child families. Students without part-time jobs were more likely to belong to moderately high-risk profiles, possibly because part-time work provides structured time management and real social interactions that can limit excessive short video immersion.

Grade level differences were not statistically significant, indicating that latent profile membership for short video addiction and emotion dysregulation may not follow a linear pattern across university years. This might suggest that the risk distribution remains relatively stable throughout the 4 years of college, or that differing developmental tasks and stressors across grades counterbalance each other’s effects on overall profile membership.

### 4.3 Practical implications

The four latent profiles identified in this study offer a more refined risk stratification framework for university mental health practitioners. Traditional single-dimension screening methods may overlook students experiencing moderate but concurrent distress in both short video addiction and emotion dysregulation, who have not yet reached clinical thresholds. Considering that Profile 3, the “Medium to upper short video addiction – medium to upper emotion dysregulation” group, comprises 44.7% of the sample, university mental health services should broaden their focus to include this large subclinical population for early prevention and monitoring. It is recommended that assessments of short video use behavior and emotional regulation difficulties be incorporated into freshmen psychological screenings, enabling risk-based tiered management.

The results provide evidence-based guidance for developing differentiated, targeted intervention programs. For high-risk students (Profile 4), comprehensive interventions should simultaneously focus on self-control over short video use and systematic enhancement of emotion regulation skills. Given the significant protective role of cognitive reappraisal, cognitive-behavioral therapy (CBT)-based reappraisal training may serve as a core component to help students identify and modify irrational cognitive patterns related to short video use and emotional experiences. To address the risk posed by emotional loneliness, interventions should integrate interpersonal relationship building and social support enhancement through group counseling, peer support, and intimate relationship skills training to alleviate loneliness. For moderate-risk groups (Profiles 2 and 3), primary prevention strategies combining mental health education and self-help resources are advisable to raise awareness of potential risks from short video use and teach basic emotion regulation techniques.

Male students, rural-origin students, and those with part-time job experience are more likely to belong to high-risk profiles; thus, university mental health services should proactively allocate more resources and tailored support to these groups. For male students, interventions should be tailored to their psychological characteristics and preferences, aiming to break down traditional gender norms that inhibit emotional expression. For rural-origin students, orientation programs could include digital literacy training and media usage guidance to promote healthy short video habits. At the environmental level, universities might collaborate with short video platforms to develop health usage reminders and time management tools targeted at student users. Simultaneously, enriching campus cultural activities, creating diverse real social opportunities, and fostering supportive peer environments can provide alternative sources of gratification, reducing the temptation to overuse digital media.

Therefore, first, strengthening adolescents’ digital/media literacy (e.g., evaluating online content, privacy protection, and self-regulation skills) is recommended, as media literacy interventions have shown beneficial effects on youths’ media-related outcomes and can reduce vulnerability to problematic online experiences (Jeong et al., 2012). Second, providing family- and school-based guidance on media use (e.g., collaborative rule-setting, active mediation, and monitoring) may help reduce risk exposure and support healthier routines, consistent with evidence that parental mediation and structured guidance are associated with safer and more adaptive digital engagement (Livingstone and Helsper, 2008; Nation et al., 2003). Third, for higher-risk profiles, multi-component approaches that combine behavioral skills training, mental health support, and targeted prevention of online risks may be warranted, consistent with broader prevention frameworks emphasizing skills-based and context-sensitive interventions for adolescent risk behaviors (Skeen et al., 2019).

### 4.4 Limitations and future directions

This study used a cross-sectional design, which can only reveal associations and latent profile distributions at a single point in time, limiting causal inference and the ability to track dynamic changes in profile membership. The relationship between short video addiction and emotion dysregulation may be bidirectional, and static cross-sectional analyses cannot capture this dynamic interplay. Future research should adopt longitudinal designs and methods such as latent transition analysis to examine profile shifts over time and their predictors, clarifying the temporal sequence and interaction mechanisms between these two dimensions.

We excluded students who were currently receiving psychiatric medication or psychological therapy. Although this decision reduced potential confounding attributable to treatment-related symptom changes, it may have introduced selection bias by under-representing individuals with higher clinical severity. Consequently, the prevalence and characteristics of the high-risk profile (Profile 4) reported here may be conservative, and the identified profile structure and predictors may not fully generalize to clinical or help-seeking student populations. Future studies should replicate the LPA in more inclusive samples and explicitly model treatment status (e.g., as a covariate or via multi-group/mixture approaches) to evaluate the robustness of the profile solution.

The sample was drawn from universities in Shandong Province using convenience and snowball sampling, restricting representativeness and generalizability. College students from different regions, types of institutions, and cultural backgrounds may exhibit varying patterns of short video use and emotion regulation. Future studies should expand geographic and institutional diversity, employ stratified random sampling to enhance representativeness, and explore cultural moderators of profile structure.

All variables were measured through self-report questionnaires, which may be subject to social desirability bias, recall bias, and limitations of self-awareness. Although procedural controls and Harman’s single-factor test were used to assess common method bias, inherent limitations remain. Future research should consider integrating multi-source data such as objective behavioral metrics, physiological indicators, and informant reports to enhance measurement validity and confidence in findings.

This study examined only two core psychological variables—cognitive reappraisal and emotional loneliness—as predictors of profile membership, potentially overlooking other important risk or protective factors. Variables such as impulsivity, self-control, attachment style, social support, perceived stress, and sleep quality may also relate to short video addiction and emotion dysregulation and warrant inclusion in future investigations.

Finally, this study focused exclusively on college students. Whether the findings generalize to other age groups remains to be tested. Short video use behaviors and emotion regulation characteristics may vary developmentally. Additionally, as short video platforms and user habits evolve, the stability of the identified profiles and influencing factors over time should be assessed through longitudinal and cross-cohort studies.

## 5 Conclusion

Using latent profile analysis, this study is the first to identify four distinct profiles of short video addiction and emotion dysregulation among college students: “Low short video addiction – low emotion dysregulation,” “Medium to upper short video addiction – medium to upper emotion dysregulation,” “Medium to lower short video addiction – medium to lower emotion dysregulation,” and “High short video addiction – high emotion dysregulation.” The results indicate a highly coordinated variation pattern at the individual level, suggesting a dynamic, mutually reinforcing relationship between these two constructs. Cognitive reappraisal, as an adaptive emotion regulation strategy, serves as a significant protective factor for low-risk profile membership, while emotional loneliness increases the likelihood of belonging to high-risk profiles. Additionally, male gender, rural origin, and part-time job experience are associated with higher risk profiles. These findings provide person-centered empirical evidence deepening our understanding of the complex interplay between short video addiction and emotion dysregulation, and offer scientific guidance for developing precise, differentiated mental health interventions.

## Data Availability

The raw data supporting the conclusions of this article will be made available by the authors, without undue reservation.

## Footnotes

1
https://www.credamo.com/
