# Supplementary material for: The association between short video addiction and emotion dysregulation among college students: a latent profile analysis and its influencing factors
Source: Front Psychol. 2026 Mar 13;17:1789207. doi: 10.3389/fpsyg.2026.1789207 (PMC13021641; doi:10.3389/fpsyg.2026.1789207)
Supplement: Supplementary file 1 [file Table_1.docx]

# The Association Between Short Video Addiction and Emotion Dysregulation Among College Students: A Latent Profile Analysis and Its Influencing Factors

# Supplementary Table S1

| Variables | Profile 1 (n=108)  M ± SD | Profile 2 (n=420)  M ± SD | Profile 3 (n=522)  M ± SD | Profile 4 (n=118)  M ± SD |
| --- | --- | --- | --- | --- |
| Short video addiction | 1.2556 ± 0.2579 | 2.2376 ± 0.2386 | 3.0065 ± 0.2282 | 3.7910 ± 0.3029 |
| Emotional dysregulation | 2.5025 ± 0.4226 | 2.8500 ± 0.3900 | 3.0408 ± 0.3920 | 3.4071 ± 0.4618 |
| SVA_F1 | 1.2346 ± 0.2831 | 2.1476 ± 0.3961 | 2.9693 ± 0.3653 | 3.7768 ± 0.4932 |
| SVA_F1 | 1.1944 ± 0.3563 | 2.2008 ± 0.4044 | 3.0568 ± 0.3918 | 3.8588 ± 0.4977 |
| SVA_F1 | 1.1605 ± 0.2715 | 2.1769 ± 0.3480 | 2.9789 ± 0.3352 | 3.8136 ± 0.4101 |
| SVA_F1 | 1.2716 ± 0.4492 | 2.2357 ± 0.4463 | 3.0179 ± 0.4304 | 3.7853 ± 0.4664 |
| SVA_F1 | 1.5278 ± 0.6606 | 2.4270 ± 0.5914 | 3.0096 ± 0.4814 | 3.7203 ± 0.5529 |
| ED1 | 2.3951 ± 0.4645 | 2.8212 ± 0.3942 | 3.0479 ± 0.3851 | 3.4386 ± 0.4797 |
| ED1 | 2.9321 ± 0.4764 | 2.9651 ± 0.5143 | 3.0121 ± 0.5210 | 3.2811 ± 0.4900 |
| Cognitive reappraisal | 3.0864 ± 0.5546 | 3.0575 ± 0.5819 | 2.9697 ± 0.5567 | 2.7825 ± 0.5865 |
| Emotional loneliness | 2.9230 ± 0.5655 | 2.9450 ± 0.5757 | 2.9920 ± 0.5374 | 3.2470 ± 0.5302 |

*Note: Values are observed means and standard deviations by most likely profile membership. SVA_F1–SVA_F5 denote the five dimensions of short video addiction used as indicators; ED1–ED2 denote the emotional dysregulation dimensions used as indicators.*

Supplementary Methods

Mplus estimation settings for the latent profile analysis (LPA)

The LPA models were estimated in Mplus Version 8.3 using mixture modeling (ANALYSIS: TYPE = MIXTURE) with the MLR estimator (robust maximum likelihood). To reduce the risk of local maxima, we used STARTS = 200 50 (200 initial-stage random starts and 50 final-stage optimizations) and PROCESSORS = 4. Model enumeration was evaluated using information criteria and classification diagnostics reported in Table 4, and we additionally requested the Vuong–Lo–Mendell–Rubin likelihood ratio test (TECH11) and the bootstrap likelihood ratio test (TECH14). The Mplus output indicated that the best log-likelihood value was replicated (“THE BEST LOGLIKELIHOOD VALUE HAS BEEN REPLICATED”), and model estimation terminated normally. Posterior class membership probabilities were saved (SAVE = CPROB).
